# Social media perceptions of college football performance and season length 2019–2023

**DOI:** 10.1371/journal.pone.0325840

**Published:** 2025-07-01

**Authors:** Michael L. Smith, Austin B. Berenda, Valerie Kilders, Nicole Olynk Widmar, Courtney Bir, F. Bailey Norwood, Zack Neuhofer, Lauren Bales

**Affiliations:** 1 Department of Agricultural Economics, Purdue University, West Lafayette, Indiana, United States of America; 2 Department of Agricultural Economics, Oklahoma State University, Stillwater, Oklahoma, United States of America; Università degli Studi di Milano: Universita degli Studi di Milano, ITALY

## Abstract

College Football carries a strong social, cultural, and economic importance in the United States with teams, universities, the public and others leveraging social media and the online sphere to promote and discuss events and happenings. In our study, we quantify the mentions and underlying public sentiment of online posts related to American College Football originating in the 2019–2023 seasons on social and news media in the United States. We complement this with an analysis of how team performance clustered into conference levels relates to social media data using ordinary least squares. We find the impact of the 2020 season, which had pandemic-induced schedule adjustments, was felt across the sports landscape, but impacted different conferences in diverse ways. We further observe that public sentiment during the season tends to be higher in Power Five conferences that have a lower winning percentage. Additionally, our results suggest that the COVID season corresponds to decreased mentions. The effect on sentiment is less clear, but we more generally find that the winning percentage (positively) predicts sentiment.

## 1. Introduction

North American college football has emerged as a culturally significant sport over the past century, specifically in the United States. The game has continually evolved over the decades to its current form. Long gone are the days of ‘three yards and a cloud of dust’ football [1], which was often heard over the radio, accessible only to locals in the recent mid-century. Today, college football is the most profitable National Collegiate Athletic Association (NCAA) sport [2] and shown on TV all over the country. Top matchups consistently receive over 5 million viewers in the regular season and nearly 30 million viewers in the post-season [3]. The respective TV contracts are correspondingly significant: the television contract for the 2024 college football playoffs alone exceeds $470 million and contracts with individual conferences to broadcast college football and other sports can surpass $1 billion annually [4].

In recent seasons, programs have expanded to not only focus on TV contracts, ticket sales, and merchandise sales but also embrace the synergistic opportunities provided by social media. Some hope the growth of program’s online presence can function to build and expand a brand and enhance program interaction with fans [5] but it also further adds to the proliferation of college football’s influence on society and culture.

We extend on these previous studies by employing social media listening to quantify online mentions and the underlying public sentiment related to college football during the 2019–2023 seasons. Relevant literature on the topic of social media engagement and sentiment concerning sports is not widespread. Where it does exist, it is often relegated to studying the effect of a few games in a professional sports league. For example, research into emotional contagion and solidarity in social media users commenting on NFL games finds that anger (among social media users) encourages discussion on social media, while sadness reduces one’s likelihood of discussing the outcome of a game [6]. Relatedly, previous research into the spatial and temporal relationships of tweets about professional basketball games (specifically in the NBA playoffs) has found that winning increases the relative positive percentage of posts with a positive sentiment and losing causes relatively more negative posts for a short period, but the authors did not calculate the percentage change in the relative positive posting percentage caused by a loss [7].

Similarly, also looking at the NBA, Gong et al. (2020) found that when consumers (or fans) begin to suspect a team is tanking, this is reflected on social media, which subsequently impacts game attendance [8]. “Tanking” refers to a team deliberately underperforming to improve prospects for a better draft pick in the off-season, in hopes of improving their long-run performance [9]. Specifically, they found that a higher volume of social media mentions suspecting the team of tanking, generates a lower attendance figure for the next game, but it does not generate long run effects into future seasons. The same question cannot be directly applied to collegiate teams because they have a different incentive structure as new players are attained through recruiting efforts, not a league-wide draft. Instead, when looking at college football, understanding the relationship between conference performance and social media activity requires a longer-term examination of historical data on both metrics.

We aim to fill this gap by leveraging social media listening data in conjunction with conference performance metrics to uncover patterns and trends that reveal how on-field performance translates to digital engagement and online sentiment. In addition, we examine how the public responded online to the differing strategies—resulting in varied season lengths—adopted by college football teams nationwide in response to the COVID-19 pandemic. The COVID-19 pandemic presents a natural case study for this research question as it had a major impact on professional as well as amateur sports throughout the United States and beyond.

Following the initial nationwide COVID-19 outbreak in the United States, significant and widespread closures, including schools, public buildings, and most public places, disrupted the normalcy of nearly all daily activities by March 2020. By March 9^th^, 2020 all major sporting events had ceased in the US including in the National Basketball Association (NBA), and the National Hockey League (NHL), both of whom were nearing completion of regular seasons. Additionally, Major League Baseball postponed their season start. In collegiate sports (all of which are governed by the NCAA), the women’s and men’s basketball tournaments were canceled. The most obvious result of this was reduced revenues in the form of tickets to attend games [10]. Some of these leagues were able to resume activity during 2020 and offered enhanced digital platforms to immerse fans in. These platforms often provided data analytics and player tracking, while actively leveraging social media to engage fans [11].

One of the primary effects of the pandemic on college football was a significant reduction in the season length and travel schedules in college football. While the college football playing season occurs from August to early January, the operation runs year-round [12]. Spring and summer practice are an important time for a coaching staff to develop talent, integrate new players, and recently, court transferring players while convincing current players to stay. The emergence of COVID-19 restrictions on facilities and gatherings created significant hurdles to bringing teams together for spring training [13].

In 2020, the college football season was slated to begin in late August. Each of the Power Five conferences committed to reworking their season schedules to reduce or eliminate games played against opponents outside of their conferences (known as non-conference). Power Five Conferences are defined as the Atlantic Coast Conference (*ACC*), the Big Ten (*Big Ten*), the Big 12 (*Big 12*), the PAC 12 (*PAC 12*) and the Southeastern Conference (*SEC*). The exact date of Week 0 depends on the season but generally falls around Labor Day weekend. In 2020, the Power Five conferences decided to delay the start of their seasons by varying lengths of time [14–17]. Additionally, college football has a few teams that are not members of any conference including Notre Dame University, the United States Military Academy (West Point), the University of Connecticut (UConn), and the University of Massachusetts Amherst (UMASS). Some of these “independents” delayed the start of their seasons as well.

Once the 2020 season began in College Football, some additional games were canceled due to case levels of COVID-19 on the football team rosters. Like other sports, attendance at these events was strictly controlled. As the season concluded, a post-season bowl boycott movement emerged, resulting in 39% of bowl games being canceled [18].

We leverage variation in season structures over time—comparing the 2020 season with other seasons in our dataset—as well as across the Power 5 conferences, due to their differing responses to the pandemic. We find that the impact of the 2020 season was felt across the sports landscape but impacted different conferences in diverse ways.

We extend upon previous studies of social media reactions to the COVID-19 pandemic’s impacts on sports and the intersection of public health and related social phenomena [19]. Our study can help researchers better understand the level of engagement that social and digital media users have with college football. We further provide insights into the relationship between public discourse, team performance as well as season length. This information can inform decisions by brand managers and marketers in the athletic departments of the Power Five football teams as well as beyond the confines of college football.

Furthermore, we present a combined dataset resulting from the merger of social media data, as well as on-field team performance data, which is aggregated by conference. What results is a novel dataset that can be used by researchers and students to examine public opinions of a quickly changing sports environment.

## 2. Methods

### Social media listening

To capture the public’s social media discourse about college football and assess responses to different season lengths, we employ social media listening facilitated via the Quid platform (formerly known as NetBase and then NetBase Quid). The methodology employed in our analysis follows and builds upon previous studies using social media listening. Widmar et al. (2020) hypothesized that social media sentiment can be viewed as a performance measure and Lai et al. (2023) highlighted the relationship between public sentiment towards cruises and resulting stock performance which was unique in probing public sentiment on difficult-to-quantify goods [20,21]. Similar explorations include a study of the connection between social media sentiment federal data on water quality [22]. Focusing more on the impact of specific events, other studies employing social media listening have looked at the relationship between social interactions and disaster events [23], baby formula shortages [24], as well as threats posed by mosquitoes [25].

In line with these studies, we assessed online and social mentions of topic related terms and the corresponding sentiment of the mentions across social media platforms, online news forums, blogs, etc. A mention constitutes a post about, or with reference to, a keyword or “included term” used to parameterize the searches. The research team generated inclusionary search terms such as the school name (ex. “Ohio State Football” and “Michigan Football” which would be two of the teams in the *Big Ten* conference cluster), each school’s team brand name (ex. “Buckeye Football” and “Wolverine Football”), each bowl game played in the past 4 seasons (ex. “Rose Bowl” and “Fiesta Bowl”), and key terms referring or relevant to the college football season. A complete list of schools and bowls included in the primary terms is provided in Table 1 (and the complete list of primary terms is in Appendix Table 1 in S1 File). Each keyword term was duplicated with a hashtag to account for those mentions (ex. “#BuckeyeFootball” and “#WolverineFootball” which would both be collected as part of the *Big Ten* mentions and net sentiment). Additionally, college football has a few teams who are not members of any conference. Some of these “independents” delayed the start of their seasons as well. Independents include the United States Military Academy (West Point), the University of Connecticut (UConn), and the University of Massachusetts Amherst (UMASS). The University of Notre Dame is included in our dataset as a member of the *ACC* despite playing football as an Independent most seasons. Notre Dame joined the *ACC* for the 2020 season and regularly plays *ACC* opponents during seasons as an Independent. This paper analyzes Power 5 quality teams at a conference level, thus omitting Notre Dame due to its independent status would eliminate a major fanbase, as major teams have been defined as Power 5 or Notre Dame. While Notre Dame is not officially a member of the *ACC*, they had to play only *ACC* opponents in 2020 due to COVID restrictions, and they are considered members of the *ACC* for scheduling purposes including frequent play against *ACC* opponents, and the ability to qualify for Bowl Games under bids reserved for the *ACC* [26,27].

**Table 1:** List of schools searched with conference groupings included.

ACC	Big Ten	Big 12	PAC 12	SEC
Clemson University	University of Illinois	Baylor University	University of Washington	University of Alabama
University of Miami	Indiana University	Iowa State University	University of Oregon	University of Arkansas
University of North Carolina	University of Iowa	University of Kansas	Stanford University	Auburn University
North Carolina State University	University of Maryland	Kansas State University	Oregon State University	University of Florida
Boston College	University of Michigan	University of Oklahoma	University of California, Berkeley	University of Georgia
University of Pittsburgh	Michigan State University	Oklahoma State University	Washington State University	Louisiana State University
Virginia Tech University	University of Minnesota	Texas Christian University	University of Southern California	University of Mississippi
University of Virginia	University of Nebraska	University of Texas	University of Colorado	Mississippi State
Wake Forest University	Northwestern University	Texas Tech University	University of Utah	University of Kentucky
Georgia Tech University	Ohio State University	West Virginia University	UCLA	University of Missouri
University of Louisville	Pennsylvania State University		Arizona State University	University of South Carolina
Florida State University	Purdue University		University of Arizona	University of Tennessee
Duke University	Rutgers University			Texas A&M
Syracuse University	University of Wisconsin			Vanderbilt University
University of Notre Dame				

It is common when using social media listening that some posts are returned that match a keyword, but with a different meaning. As such, researchers must tune the web scraper to only include the intended meaning of the keywords. Tuning is completed by excluding terms. A list of excluded terms is provided in Appendix Table 2 in S1 file. This list was developed by examining data results that are not relevant to the intent of the search. These terms relate to other versions of football including football in primary school, professional football, and the sport of soccer, which is widely known as fútbol.

A series of thematic sub-searches, which allow the parsing out of mentions and sentiment related to specific topics, were conducted. A sub-search to examine each of the Power Five Conferences, which are the top performers in the sport, was conducted to better understand how online and social media developed about each of the major conferences. Moreover, doing so also allows us to test whether there are differences in online and social media engagement and tone of conversation depending on the difference in season length, which was determined by the conference a team played in. Each conference sub-search uses team names in the same format as the primary search.

Quid quantifies mentions about a topic, but also analyzes the tone, or positivity versus negativity, of the search results (sentiment). The sentiment is summarized via a sentiment score that represents the ratio of positive relative to negative mentions. As such it can be expressed as


$$S_{t}=\frac{Positive\;Mentions_{t}-Negative\;Mentions_{t}}{Positive\;Mentions_{t}+Negative\;Mentions_{t}}\times 100$$

where $S_{i,t}$ expresses the net sentiment in week t. As a ratio it is bounded between +100 (only positive posts) and −100 (only negative posts). Whether a mention is positive, negative, or neutral is determined by Quid’s proprietary Natural Language Processing (NLP) model, which assigns an individual sentiment to search results by examining the context of the inclusionary search terms. Posts can be classified as positive, negative, or neutral, building upon previous literature studying the use of NLP on social media posts about professional sports [28]. Neutral posts are not used in the calculation of net sentiment scores; these neutral posts are used in other calculations and insights including mention counts [29]. Table 2 shows the annual count of positive, negative, and neutral posts as well as sources for data collection.

**Table 2:** Sources (and counts) of data collection and counts of positive, negative, and neutral scores on sentiment of posts.

Sentiment	2019	2020	2021	2022	2023
Neutrals	6,111,911	4,815,701	5,095,304	6,082,862	7,550,205
Positives	453,381	354,452	411,804	476,033	623,236
Negatives	158,516	165,576	142,060	189,927	227,756
**Sources**	**2019**	**2020**	**2021**	**2022**	**2023**
Twitter	5,740,890	4,328,730	4,251,360	5,102,220	7,174,590
News	617,331	683,648	1,025,216	1,455,432	1,035,141
Forums	123,182	101,046	243,521	92,246	115,894
Blogs	238,307	218,168	123,239	92,053	62,340

Note that the sentiment counts are useful for calculation of net sentiment, which is a ratio of positive to negative posts (calculated weekly, figures here present counts over the playing season).

While Quid’s NLP is highly advanced, we made minor adjustments to recategorize the sentiment of certain terms which may be initially misclassified once accounting for the specific context of our search and analysis. For example, in the context of college football, terms like offensive are not necessarily negatively connotated. Thus, we reclassified such terms to better reflect the actual sentiments that were being conveyed by the posts’ authors. In total, we reclassified three terms: defensive, offensive, and upset.

Searches were conducted for the geographies of the US and US minor outlying islands, exclusively in English. The overall dates searched for this analysis included from August 18, 2019, through January 20, 2024. Weekly quantity of mentions and sentiment of search results data was collected on the 2019, 2020, 2021, and 2022 seasons on November 16–17, 2023. Data on the 2023 season was collected February 13–14, 2024 to allow for the completion of the season prior to data collection. The exact dates used for data collection for each season are detailed in Appendix Table 4 in S1 File.

### On-field conference performance data

A secondary dataset on individual team performance using the Sports Reference website (sports-reference.com) was developed. Sports Reference is a website collecting and reporting data relevant to sports in the US. We collected information about the weekly performance of the teams, game scores, game locations, dates of the games, the status of the game as conference or non-conference, and the status of the team’s opponent as Football Bowl Subdivision (FBS) or Football Championship Subdivision (FCS) (“Non-Major” in the dataset). Conferences were allocated according to how they were structured in the social media dataset. The dataset also compiled season performance as the season progressed. These weekly team performance data were aggregated at the conference level to create a breakdown of overall performance by week of each conference. Note, that the Power Five conference winning percentages may be above 50% because each team will play a handful of non-Power Five opponents per season. Non-Power Five teams are not included in this research.

The complimentary secondary data permits us to examine the relationship between on-field performance and online and social media results. Specifically, we can assess whether the quantity of sentiment of online and social media mentions exhibits a relationship to conference weekly performance.

### Statistical and econometric analysis

To assess whether mentions or net sentiment reveals a significant relationship to conference weekly performance, we employed linear regression using ordinary least squares (OLS) [30]. The baseline models are specified as:


$$M_{i,t}=\;\alpha_{1}+\;\beta_{3}{COVID}_{t}+\beta_{4}W_{t,i\;}+\;\upsilon_{i,t}$$ (1)

$$S_{i,t}=\;\alpha_{1}+\;\beta_{1}{COVID}_{t}+\beta_{2}W_{t,i\;}+\;\varepsilon_{i,t}$$ (2)

where $S_{i,t}$ and $M_{i,t}\;$ correspond to the net sentiment and mentions in conference *i* in week *t*, respectively. $COVID_{t}$ is a dummy variable equal to 1 if week *t* falls into the 2020 regular season (and the accompanying post-season), and 0 otherwise. $W_{t,i}$ is the winning percentage of the conference in the given week. Including this variable allows us to test the relationship between on-field performance and social media conversations. Lastly, $\varepsilon_{i,t}$ and $\upsilon_{i,t}$ are error terms for sentiment and mentions, respectively. For the model which includes all conferences, we include robust standard errors, assuming that these would cluster at the conference level. We also isolate mentions and sentiment of each conference and use their on-field performance as a predictor term (along with the COVID-19 dummy variable).

To control for unobserved, time-invariant characteristics of each conference we further estimate expanded OLS regressions (equations 3 and 4) that include dummy variables for each of the Power Five conferences, omitting the Pac 12 as the reference category. These dummy variables capture conference-specific fixed effects, accounting for any latent, stable factors unique to each conference. Robust standard errors are computed, clustered at the conference level, to address potential intra-cluster correlation in the error terms. These expanded OLS take the form:


$$M_{i,t}=\;\alpha_{3}+\;\beta_{5}{COVID}_{t}+\beta_{6}W_{t,i\;}+\sum_{j\neq Pac12}\gamma_{j}C_{j\;}+\;\upsilon_{i,t}$$ (3)

$$S_{i,t}=\;\alpha_{4}+\;\beta_{7}{COVID}_{t}+\beta_{8}W_{t,i\;}+\sum_{j\neq Pac12}\gamma_{j}C_{j\;}+\;\varepsilon_{i,t}$$ (4)

Where γ is the vector of coefficients and *C* is the vector of dummy variables *j* through *n* and the other variables are the same as they were in equations (1) and (2).

While fixed effects control for unobserved heterogeneity across conferences, the structure of our dataset – observations nested within weeks across multiple conferences—introduces a hierarchical or multilevel structure. Specifically, each calendar week is observed repeatedly across all conferences.

The repeated nature of the data precludes formal declaration of time series, making tests like the Durbin-Watson test untenable. To account for potential week-level unobserved heterogeneity and correlation among observations that share the same week, we estimate multilevel mixed-effects models with random intercepts for weeks. This approach allows for a unique baseline level for each week, capturing shared shocks or temporal patterns that affect all conferences during a given week. These mixed-effects models take the form:


$$M_{i,t}=\;\alpha_{5}+\;\beta_{9}{COVID}_{t}+\beta_{10}W_{t,i\;}+u_{t}+\;\upsilon_{i,t}\;$$ (5)

$$S_{i,t}=\;\alpha_{6}+\;\beta_{11}{COVID}_{t}+\beta_{12}W_{t,i\;}+u_{t}+\;\varepsilon_{i,t}$$ (6)

Where $u_{t}$ is a random intercept for an index of weeks and each other parameter remains as they were before.

Lastly, to compare average weekly mentions and sentiment between COVID-affected and non-COVID seasons, we conduct two-tailed t-tests assuming unequal variances. Recall, that the 2020 season was shortened and had no inter-conference play until the postseason. This comparison is conducted for the national dataset, as well as the Power Five conference individual datasets. The timeline studied began the week of the first college football game which is generally around the 3^rd^ full week of August. Due to the high cost of collecting social media datasets specific to each individual team, we analyze playing performance and social media activity at the conference level. We contribute the attached dataset to provide opportunities for future analysis and classroom exercises which could entail analysis of individual team performance on their conference social media data or collecting social media data at the team level.

## 3. Results and discussion

### Descriptive results

Table 3 displays the total and average mentions and sentiment broken down by conference, only mentions occurring during the playing season were included. The *SEC* and the *Big Ten* dominate each season in terms of mentions. Rankings of the highest sentiment conferences vary by season, but we see that over the 5 season the *Big 12* and the *Pac 12* have the highest sentiment, indicating the highest ratio of positive to negative posts. The *Big Ten* and the *SEC* sentiment scores rank 4^th^ and 5^th^ over the 5 seasons, albeit, still with a positive sentiment score of around 37%. Curiously, this suggests the most successful conferences have the highest number of mentions but also the lowest weekly sentiment. This could be because it is difficult to appropriate sentiment, especially in comments mentioning multiple teams. Effectively, a fan might love watching their team win, as much as they love watching another team lose. Such metrics could be a result of fans from less successful conferences expressing their disdain for the *Big Ten* and *SEC*. Additionally, this might suggest the games elicit strong levels of emotion due to the nature of the game.

**Table 3:** Rankings of top mentions and sentiment by power five conference.

Total Mentions		Average Sentiment		Average Mentions	
Conference	Total Mentions	Conference	Avg Sentiment	Conference	Avg Mentions
SEC	1,703,004	Big 12	45.5	SEC	15,111.5
Big Ten	1,622,961	Pac 12	44.4	Big Ten	14,327.0
Big 12	1,077,364	ACC	41.3	Big 12	9,537.1
Pac 12	1,058,771	Big Ten	37.8	Pac 12	9,429.3
ACC	900,768	SEC	36.8	ACC	7,983.6

Averages are based on weekly data figures only including the playing season (dates are described in Table 6).

Looking at performance on the field, we find the *SEC* had the highest winning percentage in 4 out of the 5 seasons (Appendix Table 3 in S1 File). The *Big Ten* was the Second-place finisher in 3 of the 5 seasons and the *PAC 12* consistently ranked last in yearly conference winning percentage. However, each conference except the *PAC 12* had a seasonal winning percentage above 50% and the *PAC 12* is rarely far below 50%. This makes sense given the fact that most games played during the season are against conference opponents. It can be inferred that each conference will have a winning percentage of 50% for in-conference games. The *Big Ten*, *Big 12*, and *Pac 12* all generally play 9 in-conference games to be played by every team during the season, while the *SEC* and *ACC* generally play 8. If each *Big Ten* team plays 9 conference games, 126 *Big Ten* conference games will be contested, and each game will yield a winner and a loser. Thus, each conference has a winning percentage of around 50%. Deviations from 50% are determined by non-conference games. The more non-conference games that are won by individual teams, the higher the conference’s total winning percentage will be.

### Statistical results

Table 4 presents the results of the OLS model, analyzing weekly mentions and sentiment related to college football and teams affiliated with individual conferences over a four-year period. Looking first at the National data we find no statistically significant relationship between conference winning percentage or the COVID indicator for either net sentiment or mentions. The same holds for the *ACC* conference.

However, results for the other conferences are more mixed. We find a statistically significant positive relationship between net sentiment and weekly winning percentage for the *Big 12, Big Ten, Pac 12*, and *SEC*, with the strongest response observed for the *SEC*. In contrast, the COVID variable is negatively and significantly associated with net sentiment for the *Big 12, Big Ten,* and *Pac 12*. These results could suggest that fans are marginally more passionate in reaction to a win in the SEC, or that fans who comment on SEC and ACC games cared less about COVID-19 (possibly because their conferences decided to start the playing season earlier than the other conferences). Turning to mentions, COVID is negatively associated with weekly mentions at a statistically significant level (at least at the 10% level) for all conferences except the *ACC*. Additionally, weekly winning percentage is positively associated with mentions for the *Big 12, Big Ten*, and *Pac 12*.

**Table 4:** OLS results (with standard errors).

	National	ACC	Big 12	Big Ten	Pac 12	SEC
**Mentions**						
Weekly winning %	4,092.44	3,174.93	6,885.28**	10,359.55**	6,885.278**	−1,259.14
	(2,121.65)	(2,489.21)	(2,601.37)	(5,515.57)	(2,601.37)	(3,135.49)
COVID	−2,431.59	−1,459.16	−3,413.35**	−4,327.39*	−3,413.35**	−3,993.94**
	(1,253.691)	(1,003.13)	(1,342.53)	(2,206.78)	(1,342.53)	(1,434.34)
$\upsilon_{i,t}$	10,547.39**	7,314.8**	7,600.29**	11,530.42**	7,600.3**	16,838.4**
	(1,002.46)	(1,458.4)	(1,503.27)	(3,262.56)	(1,503.27)	(1,928.97)
**Sentiment**						
Weekly winning %	24.18	24.8	21.68**	36.19**	21.68**	59.97**
	(11.34)	(16.66)	(10.5)	(17.56)	(10.50)	(14.44)
COVID	−8.28	−8.92	−9.71*	−21.27**	−9.71*	4.83
	(4.02)	(6.71)	(5.42)	(7.02)	(5.42)	(6.61)
$\varepsilon_{i,t}$	25.18**	23.7	32.44**	17.37*	32.44**	−2.28
	(3.89)	(9.76)	(6.07)	(10.39)	(6.07)	(8.89)

** *and*

* *denote significance at the 5% and 10% level, respectively*.

Table 5 shows results from the expanded OLS model for sentiment and mentions. Omitting the *Pac 12* dummy variable requires that we interpret conference fixed effects against the *Pac 12* as a baseline. From this viewpoint, net sentiment is lower in the *Big Ten, ACC,* and *SEC*, but higher in the *Big 12*. We observe that at the 10% significance level, an increased winning percentage has a positive effect on net sentiment. Similarly, we do not observe that the COVID-19 2020 season is an accurate predictor of changes in net sentiment.

**Table 5:** Expanded OLS with dummy variables for Mentions and Net Sentiment at the Conference level.

	Coefficient	Robust Standard Error
**Mentions**		
Weekly winning %	2,687.23	2,497.28
COVID	−2,354.41*	1,105.1
Big Ten	6,072.69***	103.83
Big 12	209.55*	78.32
ACC	−1,703.64***	87.3
SEC	4,803.68***	138.51
Intercept	9,460.51***	1,202.83
**Sentiment**		
Weekly winning %	26.36*	11.79
COVID	−8.1	4.26
Big Ten	−6.07***	0.68
Big 12	2.58**	0.63
ACC	−4.34***	0.72
SEC	−7.57***	0.99
Intercept	27.03**	6.19

*** *,*

** *and*

* *denote significance at the 1%, 5% and 10% level, respectively*.

Examining results concerning mentions, we see that the conference level fixed effects have strong levels of statistical significance. Relative to the *PAC 12*, we see the *Big Ten* and *SEC* have considerably higher levels of weekly mentions, while the *Big 12* coefficient on mentions is very close to 0. The *ACC* variable reflects roughly 1700 fewer mentions per week compared to the *Pac 12*. We do not find evidence that weekly winning percentage is a strong predictor of mentions in the expanded model. However, the COVID-affected 2020 season is associated with a statistically significant reduction in mentions, indicating lower online fan engagement during that period. This effect is significant at the 10% level in the expanded OLS model, whereas it was significant at the 5% level in the reduced-form OLS specification.

Results from equations (5) and (6), shown in Table 5, a few notable takeaways emerge. When predicting conference mentions, our intercept gives us a baseline count of mentions we could expect to see if all other variables are equal to zero. At a high level of statistical significance, we see the 2020 season, which was impacted by COVID-19, saw considerably fewer mentions of the teams during the college football season. We do not find evidence that better on-field performance translates to higher levels of engagement (measured in mention count) on social media. Examining the random effects, we observe high levels of variations across weeks of the season and high levels of unexplained variance which suggests additional factors at play in the data (discussed further in the limitations).

When predicting conference sentiment with mixed effects, shown in Table 6, the model intercept provides a useful baseline, indicating that net sentiment is generally positive, with an average of +26 The results show that weekly winning percentage has a strong positive effect on net sentiment, suggesting that better on-field performance is associated with more favorable fan reactions. Additionally, the 2020 season—disrupted by COVID-19 and characterized by a shortened schedule and limited inter-conference play—was associated with significantly lower net sentiment. The residual variance on Conference net sentiment was 23.1. Considering the possible range of net sentiments [−100,100], this indicates substantial unexplained variation remains in the model. This underscores the complexity of factors that shape online sentiment beyond just performance and season structure.

**Table 6:** Mixed effects models of mentions and net sentiment at the conference level.

	Sentiment	Mentions
**Fixed Effects**		
Weekly winning %	22.39*** (6.81)	2,672.61 (1,768.46)
COVID	−8.10** (3.14)	−2,343.91*** (805.25)
Intercept	26.39*** (4.03)	11,321.36*** (1,058.24)
**Random Effects**		
Week	3.35	1,143.65
Residual	23.10	5,906.86

*** *,*

** *and*

* *denote significance at the 1%, 5% and 10% level, respectively*.

More succinctly, we find that engagement is positive in season length and winning percentage. This shows the public reaction to the 2020 season (which saw fewer games and almost no inter-conference play) resulted in lower engagement (in mentions) and lower sentiment (among those who did engage) on social media. These findings suggest that having a winning team may generate positive knock-off effects for an athletic department and conference brand.

However, it would be difficult to isolate the effects of season length and performance from the broader context of the pandemic, as the unique circumstances of COVID-19 are not easily replicable. Ultimately, the extent to which athletic departments can leverage these dynamics for storytelling, brand development, and fan engagement depends on the level of social media interaction—interaction that, according to our findings, is closely tied to both the quality (i.e., success) and quantity (i.e., number of games) of on-field performances.

Many of our coefficients are statistically significant and interpretable. Comparing conferences, movement in sentiment and mentions is explained in the *Big Ten* and CFB altogether, however we lack statistical significance to explain these movements in the *ACC*. It is difficult to read into what this variation in statistical significance between conferences may suggest. By the nature of our combined dataset, with limited games per season, it is possible that the difference in sample size and timing could generate different findings. Alternatively, these findings might make sense, in the *ACC* and *PAC 12*, there may be a lower level of average general football interest among the community.

To further analyze the effect of season length on fan engagement, we construct a T-test comparing the shortened season to the four other seasons collected. The results of the T-test of mentions and sentiment are shown in Tables 7 and 8, respectively. Taken together, we see the national dataset exhibits a significant difference in both sentiment and mentions between 2020 and non-2020 seasons (based on the two-tail result). In both cases, the national data set showed a decline in both the quantity of attention and sentiment of the associated media for the 2020 shortened season. Effectively, this can be interpreted as a decline in the national level of engagement during the 2020 season. Results by conference are mixed, however. We find a significant difference between mentions in the *Big Ten*, *Big 12*, and the *SEC*. In these conferences, alterations to the season had an impact on fan engagement. In those three cases, mentions were lower. However, only the *Big 12* exhibits significant differences between sentiments, which also fell in 2020. These differences suggest that the varying approaches taken by each conference to managing the 2020 season—such as start dates, number of games, and policies on inter-conference play—may have influenced how fans engaged with and emotionally responded to their teams online.

**Table 7:** Results of T-test of two samples differing by season length. Assuming unequal variances.

Units are count of Mentions												
	Big Ten		Big 12		Pac 12		SEC		ACC		National	
	Non-2020 Season	2020 Season	Non-2020 Season	2020 Season	Non-2020 Season	2020 Season	Non-2020 Season	2020 Season	Non-2020 Season	2020 Season	Non-2020 Season	2020 Season
Mean	15,508.7	10,079.3	10,100.8	7,532.8	9,333.0	9,974.9	15,829.8	12,499.7	8,335.3	6,774.1	229,155.8	149,015.4
Variance	4.98E + 07	1.38E + 07	2.78E + 07	3.82E + 06	2.49E + 07	3.48E + 07	2.98E + 07	1.29E + 07	1.34E + 07	1.02E + 07	1.25E + 10	2.50E + 09
Observations	91.0	21.0	91.0	21.0	91.0	21.0	91.0	21.0	91.0	21.0	91.0	21.0
df	58.0		88.0		27.0		44.0		33.0		72.0	
t Stat	4.949		3.677		−0.462		3.435		1.960		5.002	
P(T<=t) one-tail	0.000		0.000		0.324		0.001		0.029		0.000	
t Critical one-tail	1.672		1.662		1.703		1.680		1.692		1.666	
P(T<=t) two-tail	0.000		0.000		0.648		0.001		0.058		0.000	
t Critical two-tail	2.002		1.987		2.052		2.015		2.035		1.993	

Seasons Tested: 2019, 2020, 2021, 2022, 2023

Hypothesized mean difference = 0

**Table 8:** Results of the T-test for two samples differing by season length. Assuming unequal variances.

Units are Net Sentiment												
	Big Ten		Big 12		Pac 12		SEC		ACC		National	
	Non-2020 Season	2020 Season	Non-2020 Season	2020 Season	Non-2020 Season	2020 Season	Non-2020 Season	2020 Season	Non-2020 Season	2020 Season	Non-2020 Season	2020 Season
Mean	39.7	29.7	48.0	36.0	44.3	45.0	37.3	36.0	43.3	34.0	46.8	35.5
Variance	507.5	855.4	427.1	585.7	710.7	566.7	750.6	381.5	590.9	556.0	153.8	249.5
Observations	91.0	21.0	91.0	21.0	91.0	21.0	91.0	21.0	91.0	21.0	91.0	21.0
df	26.0		27.0		33.0		40.0		31.0		26.0	
t Stat	1.478		2.104		−0.127		0.257		1.609		3.059	
P(T<=t) one-tail	0.076		0.022		0.450		0.399		0.059		0.003	
t Critical one-tail	1.706		1.703		1.692		1.684		1.696		1.706	
P(T<=t) two-tail	0.151		0.045		0.900		0.799		0.118		0.005	
t Critical two-tail	2.056		2.052		2.035		2.021		2.040		2.056	

Seasons Tested: 2019, 2020, 2021, 2022, 2023

Hypothesized mean difference = 0

## 4. Conclusion

We examine and quantify public engagement with US college football at a unique time in the history of the sport. The game and the league of teams have seen considerable changes in the past few seasons including the emergence of the COVID-19 pandemic which impacted the 2020 playing season. Using social media listening techniques, we assess the volume and sentiment of public discourse surrounding college football, with a particular focus on the Power Five conferences. We relate this engagement to two key factors: the effects of the COVID-19 season and each conference’s winning percentage.

Our results reveal that, at the individual conference level, higher winning percentages are associated with increased mentions and more favorable net sentiment. Looking at the aggregated data, we similarly observe a positive effect of winning percentage on net sentiment across both the expanded OLS model, which includes conference-level fixed effects, and our mixed-effects model. However, this pattern does not hold at the aggregate level where the winning percentage does not significantly predict the volume of mentions in either model.

Both models do show that the COVID-19 season is associated with a decline in engagement, as measured by mentions. The impact of the COVID-19 season on sentiment, however, is mixed. The fixed effects model does not identify it as a significant predictor, while the mixed-effects model suggests it led to decreased sentiment among online users. For the individual Power Five conferences we saw a varied impact of the 2020 COVID-19 season. While sentiment remained largely unchanged—with the exception of the *PAC 12*—the shortened season notably decreased mentions in the *Big Ten, Big 12*, and *SEC*.

These findings offer several insights. First, winning improves online sentiment, a valuable insight for athletic brand managers aiming to anticipate and shape public engagement. Particularly, considering the NCAA rule change towards allowing college athletes to profit from their Name, Image, and Likeness (NIL), social media attention has grown in importance towards marketing and profiting as young talent. Indeed, in a survey of 1,100 Division I athletes, it was found that 72% of commercial activity by college athletes falls into the social media category [31].

Additionally, this relationship offers a compelling case study for educational contexts, where students can test and refine such findings using novel datasets. Second, the observed decline in online engagement during the COVID-19 season highlights the importance of gameplay and season continuity in maintaining fan interest. This suggests that playtime is important to encourage fan interaction, and reduced season lengths will not help engage fans in the sport.

### Limitations and avenues for future research

Our study is subject to several limitations and, importantly, opens several promising avenues for future research. First, online media data is aggregated at the conference level. While it is costly to capture, and complex to communicate, future research could examine the mention counts and net sentiment of each team along with each team’s respective on-field performance figures.

Secondly, intra-seasonal (weekly) fluctuations in the number of games played within a conference (regardless of the opponent being in or out of the conference) might be hypothesized to influence mentions and net sentiment in the same week (and possibly the week after). Our model, which posits mention counts and net sentiment as a function of winning percentage and the existence of COVID protocols, does not account for whether there was a game in time t not the number of games played. However, the winning teams get talked about whether they play that week or not a lot of the time because of the way rankings are discussed- which is proxied by the winning percentage Subsequent studies could establish a finer temporal resolution and restructure their dataset to be able to account for such short-term dynamics.

Across models, we receive repeated indications that there is unexplained variation. The introduction of additional predictor terms could enhance the efficiency of our models. Some additional variables that may help would be a dummy variable for rivalry games, upsets (unexpected losses), and a variable reflecting the number of people watching a game in person, on TV, and radio, among other possibilities. The nature of our dataset precludes robust examination of temporal dependencies that one would expect in time-varying social media data. Two-way clustered standard errors would account for within-conference correlation as well as within-week correlations, but these are untenable without transformation because the week index is not perfectly nested into conferences. Alternatively, generating lagged mentions and sentiment variables could be useful for detecting temporal relationships that persist over time. However, the structure of the dataset would require transformation to avoid pushing lags for one conference into the lagged series of another. We thus encourage future studies to further examine this issue and build upon the dataset generated for this study.

Methodologically, our dataset’s structure limits the ability to model time-varying dynamics. The hierarchical and nested nature of the data—teams within conferences, games within weeks—poses challenges for traditional time-series or panel analysis. For example, two-way clustered standard errors (e.g., by week and conference) would be ideal to address potential correlations within groups but are infeasible here due to imperfect nesting of time periods across conferences. Likewise, generating lagged versions of engagement or sentiment measures could illuminate temporal dependencies, but such transformations risk contamination between conference-specific series unless the data are carefully segmented. A future line of work could involve restructuring the dataset into a more formal panel format, enabling richer temporal modeling techniques, such as autocorrelation tests or dynamic panel regression models.

Finally, future studies may replicate our findings in other collegiate sports or professional leagues, allowing for comparisons across athletic domains. They may also consider using open-weight or open-source NLP models for sentiment scoring to increase replicability and transparency. While our use of Quid offers access to licensed social media data and NLP pipelines, we recognize the increasing emphasis on open science, as discussed by Abdurahman et al. [32]. We encourage the development of complementary studies using reproducible, open-source frameworks to validate and extend our results.

## Supporting information

S1 File Appendix. (DOCX)

S2 File Raw data used in the analyses presented in the manuscript. This Excel file contains multiple sheets: a README sheet; a Time Series sheet with conference-level performance, online media mentions, and net sentiment; and additional sheets showing annual game-level performance by conference.
(XLSX)
