# Supplementary material for: Social media perceptions of college football performance and season length 2019–2023
Source: PLoS One. 2025 Jul 1;20(7):e0325840. doi: 10.1371/journal.pone.0325840 (PMC12212522; doi:10.1371/journal.pone.0325840)
Supplement: S1 File — (DOCX) [file pone.0325840.s001.docx]

**Appendix/Supporting Information**

AT1: Full list of terms: College football, #Collegefootball, Heisman, #Heisman, FBS, #FBS, NCAA football, #NCAAFootball, American Athletic Conference football, Atlantic Coast Conference football, ACC football, #ACCFootball, Atlantic Coast football, #AtlanticCoastFootball, Big 12 football, #Big12Football, Big 10 Football, #Big10Football, Pac-12 football, Pac 12 football, #Pac12Football, Southeastern Conference football, #SoutheasternConferenceFootball, SEC football, #SECFootball, Rose Bowl, #RoseBowl, Sugar Bowl, #SugarBowl, Cotton Bowl Classic, #CottonBowlClassic, Peach Bowl, #PeachBowl, Fiesta Bowl, #FiestaBowl, Orange Bowl, #OrangeBowl, Myrtle Beach Bowl, #MyrtleBeachBowl, Montgomery Bowl, #MontgomeryBowl, Famous Idaho Potato Bowl, #FamousIdahoPotatoBowl, Boca Raton Bowl, #BocaRatonBowl, New Orleans Bowl, #NewOrleansBowl, New Mexico Bowl, #NewMexicoBowl, Carmellia Bowl, #CarmelliaBowl, #FirstResponderBowl, Lending Tree Bowl, #LendingTreeBowl, Cure Bowl, #CureBowl, Cheez-It Bowl, #CheezitBowl, Alamo Bowl, #AlamoBowl, Armed Forces Bowl, #ArmedForcesBowl, Arizona Bowl, #ArizonaBowl, Liberty Bowl, #LibertyBowl, Citrus Bowl, #CitrusBowl, Gator Bowl, #GatorBowl, Outback Bowl, #OutbackBowl, College Football Playoff, #AlabamaFootball, Crimson Tide football, #CrimsonTideFootball, Razorback football, #RazorbackFootball, #AuburnFootball, Tigers football, #TigersFootball, #FloridaFootball, Gator Football, #GatorFootball, #GeorgiaFootball, Bulldog Football, #BulldogFootball, Wildcat football, #WildcatFootball, LSU Football, #LSUFootball, Tiger football, #Tigerfootball, Ole Miss football, #OleMissFootball, Mississippi State football, #MississippiStateFootball, Bulldog football, #MissouriFootball, #SouthCarolinaFootball, Gamecock football, #GamecockFootball, Volunteer football, #VolunteerFootball, Texas A&M football, #TexasA&MFootball, Aggie football, #AggieFootball, Vanderbilt football, #VanderbiltFootball, Commodore football, #CommodoreFootball, #IllinoisFootball, Fighting Illini football, #FightingIlliniFootball, Indiana University, #IndianaUniversity, Hoosier football, #HoosierFootball, #IowaFootball, Hawkeye football, #HawkeyeFootball, Terrapin football, #TerrapinFootball, #MichiganFootball, Wolverine football, #WolverineFootball, Michigan State University football, #MichiganStateFootball, Spartan football, #SpartanFootball, #MinnesotaFootball, Golden Gopher football, #GoldenGopherFootball, #NebraskaFootball, #OhioStateFootball, Buckeye football, #BuckeyeFootball, Penn State Football, #PennStateFootball, Nittany Lion football, #NittanyLionFootball, #PurdueFootball, Boilermaker football, #BoilermakerFootball, Rutgers football, #RutgersFootball, Scarlet Knight football, #ScarletKnightFootball, #WisconsinFootball, Badger football, #BadgerFootball, Baylor football, #BaylorFootball, #BearFootball, Iowa State football, #IowaStateFootball, Cyclone football, #CycloneFootball, Jayhawk football, #JayHawkFootball, Kansas state football, #KansasStateFootball, Sooner football, #SoonerFootball, Cowboy football, #CowboyFootball, TCU football, #TCUFootball, Horned Frogs football, #HornedFrogsFootball, Longhorn football, #LonghornFootball, Texas Tech football, #TexasTechFootball, Red Raider Football, #RedRaiderFootball, West Virginia football, #WestVirginiaFootball, Mountaineer football, #MountaineerFootball, Washington football, #WashingtonFootball, Husky football, #HuskyFootball, #OregonFootball, Ducks football, #DucksFootball, Stanford football, #StanfordFootball, Cardinal football, #CardinalFootball, Oregon State football, #OregonStateFootball, Beaver football, #BeaverFootball, Golden Bears football, #GoldenBearsFootball, Washington State football, #WashingtonStateFootball, Cougar football, #CougarFootball, USC football, #USCFootball, Trojans football, #TrojansFootball, Buffalo football, #BuffaloFootball, #UtahFootball, Utes football, #UtesFootball, UCLA football, #UCLAFootball, Bruins football, #BruinsFootball, Arizona State football, #ArizonaStateFootball, Sun Devils football, #SunDevilsFootball, Arizona football, #ArizonaFootball, Notre Dame football, #NotreDameFootball, Fighting Irish football, #FightingIrishFootball, Clemson football, #ClemsonFootball, Miami football, #MiamiFootball, Hurricanes football, #HurricanesFootball, North Carolina football, #NorthCarolinaFootball, Tar Heels football, #TarHeelsFootball, NC State football, #NCStateFootball, Wolfpack football, #WolfpackFootball, Eagles football, #EaglesFootball, Pittsburgh football, Panther football, #PantherFootball, Virginia Tech football, #VirginiaTechFootball, Hokie football, #HokieFootball, Virginia football, #VirginiaFootball, Cavalier football, #CavalierFootball, Wake Forest football, Demon Deacon football, #WakeForestFootball, #DemonDeaconFootball, Georgia Tech football, #GeorgiaTechFootball, Yellow Jacket football, Louisville Football, #LouisvilleFootball, Florida State football, #FloridaStateFootball, Seminole football, #SeminoleFootball, Duke football, #DukeFootball, Blue Devils football, #BlueDevilsFootball, Syracuse football, #SyracuseFootball, Orange football, #OrangeFootball, First Responder Bowl, UCF football, #UCFfootball, Knights football, #knightsfootball, BYU football, #BYUfootball, Cincinnati football, #cincinnatifootball, Houston football, #Houstonfootball, Bearcats football, #bearcatsfootball, Cougars football, #cougarsfootball, Alabama Football, Arkansas Football, #ArkansasFootball, Auburn Football, Florida Football, Georgia Football, Kentucky Football, #KentuckyFootball, Rebels Football, #RebelsFootball, Missouri Football, South Carolina Football, Tennessee Football, #TennesseeFootball, Illinois Football, Iowa Football, Maryland Football, #MarylandFootball, Michigan Football, Minnesota Football, Nebraska Football, Cornhusker Football, #CornhuskerFootball, Northwestern Football, #NorthwesternFootball, Ohio State Football, Purdue Football, Wisconsin Football, Kansas Football, #KansasFootball, Oklahoma Football, #OklahomaFootball, Oklahoma State Football, #OklahomaStateFootball, Texas Football, #TexasFootball, Oregon Football, California Football, #CaliforniaFootball, Colorado Football, #ColoradoFootball, Utah Football, Boston Football, #BostonFootball, #PittsburghFootball, #YellowJacketFootball, Makers Wanted Bahamas Bowl, #MakersWantedBahamasBowl, Tropical Smoothie Cafe Frisco Bowl, #TropicalSmoothieCafeFriscoBowl, Celebration Bowl, #CelebrationBowl, FBC Mortgage Cure Bowl, #FBCMortgageCureBowl, Cheribundi Boca Raton Bowl, #CheribundiBocaRatonBowl, Camellia Bowl, #CamelliaBowl, Mitsubishi Motors Las Vegas Bowl, #MitsubishiMotorsLasVegasBowl, R+L Carriers New Orleans Bowl, #R+LCarriersNewOrleansBowl, Bad Boy Mowers Gasparilla Bowl, #BadBoyMowersGasparillaBowl, SoFi Hawai'i Bowl, #SoFiHawai'iBowl, Walk On's Independence Bowl, #WalkOn'sIndependenceBowl, Quick Lane Bowl, #QuickLaneBowl, Military Bowl, #MilitaryBowl, New Era Pinstripe Bowl, #NewEraPinstripeBowl, Academy Sports + Outdoors Texas Bowl, #AcademySports+OutdoorsTexasBowl, SDCCU Holiday Bowl, #SDCCUHolidayBowl, Goodyear Cotton Bowl, #GoodyearCottonBowl, Camping World Bowl, #CampingWorldBowl, Chick-fil-A Peach Bowl, #Chick-fil-APeachBowl, PlayStation Fiesta Bowl, #PlayStationFiestaBowl, SERVPRO First Responder Bowl, #SERVPROFirstResponderBowl, Franklin Amer. Mort. Music City Bowl, #FranklinAmerMortMusicCityBowl, Redbox Bowl, #RedboxBolw, Capital One Orange Bowl, #CapitalOneOrangeBowl, Belk Bowl, #BelkBowl, Tony the Tiger Sun Bowl, #TonytheTigerSunBowl, AutoZone Liberty Bowl, #AutoZoneLibertyBowl, NOVA Home Loans Arizona Bowl, #NOVAHomeLoansArizonaBowl, Valero Alamo Bowl, #ValeroAlamoBowl, Vrbo Citrus Bowl, #VrboCitrusBowl, Rose Bowl Game, #RoseBowlGame, Allstate Sugar Bowl, #AllstateSugarBowl, TicketSmarter Birmingham Bowl, #TickerSmarterBirminghamBowl, TaxSlayer Gator Bowl, #TaxSlayerGatorBowl, Lockheed Martin Armed Forces Bowl, #LockheedMartinArmedForcesBowl, LendingTree Bowl, CFP National Championship, #CFPNationalChamptioship, RoofClaim.com Boca Raton Bowl, #RoofClaim.comBocaRatonBowl, Union Home Mortgage Gasparilla Bowl, #UnionHomeMortgageGasparillaBowl, TransPerfect Music City Bowl, #TransPerfectMusicCityBowl, Offerpad Arizona Bowl, #OfferpadArizonaBowl, Mercari Texas Bowl, #MercariTexasBowl, Captial One Orange Bowl, Bahamas Bowl, #BahamasBowl, Tailgreeter Cure Bowl, #TailgreeterCureBowl, Cricket Celebration Bowl, #CricketCelebrationBowl, PUBG Mobile New Mexico Bowl, #PUBGMobileNewMexicoBowl, Radiance Technologies Independence Bowl, #RadianceTechnologiesIndependenceBowl, Jimmy Kimmel LA Bowl, #JimmyKimmelLABowl, Frisco Football Classic, #FriscoFootballClassic, EasyPost Hawai'i Bowl, #EasyPostHawai'iBowl, TaxAct Camellia Bowl, #TaxActCamelliaBowl, Guaranteed Rate Bowl, #GuaranteedRateBowl, Wasabi Fenway Bowl, #WasabiFenwayBowl, SRS Distribution Las Vegas Bowl, #SRSDistributionLasVegasBowl, TaxAct Texas Bowl, #TexActTexasBowl, HomeTown Lenders Bahamas Bowl, #HomeTownLendersBahamasBowl, Duluth Trading Cure Bowl, #DuluthTradingCureBowl, Frisco Bowl, #FriscoBowl, Bad Boy Mowers Pinstripe Bowl, #BadBoyMowersPinstripeBowl, Barstool Sports Arizona Bowl, #BarstoolSportsArizonaBowl, Vrbo Fiesta Bowl, #VrboFiestaBowl, ReliaQuest Bowl, #ReliaQuestBowl, Cheez-It Citrus Bowl, #Cheez-ItCitrusBowl, Duke's Mayo Bowl, #DukesMayoBowl, Avocados From Mexico Cure Bowl, #AvacadosFromMexicoCureBowl, Isleta New Mexico Bowl, #IsletaNewMexicoBowl, LA Bowl, #LABowl, Famous Toastery Bowl, #FamousToasteryBowl, Scooter's Coffee Frisco Bowl, #ScootersCoffeeFriscoBowl, Birmingham Bowl, #BirminghamBowl, 68 Ventures Bowl, #68VenturesBowl, DirecTV Holiday Bowl, #DirecTVHolidayBowl, Pop-Tarts Bowl, #PopTartsBowl

Appendix Table 2: List of Excluded Terms.

| Excluded Terms | |
| --- | --- |
| Sunday Night Football | High School |
| NFL | pee wee |
| #NFL | little league |
| superbowl | touch football |
| super bowl | flag football |
| #superbowl | Turning Point USA |
| National Football League | realmadrid |
| Jr. High | Washington Football Team |
| Junior High | Border Patrol |
| Middle School | |

Appendix Table 3**:** Total Games Played by the Power Five Conferences in FBS Football.

| **Conference** | Total games played by season (winning %) | | | | |
| --- | --- | --- | --- | --- | --- |
|  | 2019 | 2020 | 2021 | 2022 | 2023 |
| **ACC** | 194  (56.7%) | 159  (51.6%) | 194  (57.2%) | 195  (56.4%) | 198  (57.1%) |
| **Big Ten** | 176  (57.4%) | 105  (50.5%) | 185  (57.8%) | 182  (56.6%) | 183  (57.9%) |
| **Big 12** | 129  (55.8%) | 110  (53.6%) | 130  (56.9%) | 132  (56.8%) | 183  (55.7%) |
| **Pac 12** | 154  (55.2%) | 65  (49.2%) | 153  (47.1%) | 156  (54.5%) | 156  (57.7%) |
| **SEC** | 181  (59.7%) | 151  (51.7%) | 183  (60.7%) | 185  (63.2%) | 181  (60.2%) |
| **Total** | 834  (57.1%) | 590  (51.5%) | 845  (56.2%) | 850  (57.6%) | 901  (57.7%) |

Appendix Table 4**:** Date Ranges used for each Season for statistical comparison.

| Season | Season Start | CFB Title Game | Data Period End |
| --- | --- | --- | --- |
| 2019 | 24-Aug-19 | 13-Jan-20 | 19-Jan-20 |
| 2020 | 3-Sep-20 | 11-Jan-21 | 10-Jan-21 |
| 2021 | 28-Aug-21 | 10-Jan-22 | 16-Jan-22 |
| 2022 | 27-Aug-22 | 9-Jan-23 | 15-Jan-23 |
| 2023 | 26-Aug-23 | 8-Jan-24 | 28-Jan-24 |

Appendix Table 5**:** Visualization of Results Clusters from T Test.

| **Significant Difference Groups** | |  | **No Difference Groups** | |
| --- | --- | --- | --- | --- |
| **Sentiment** | **Mentions** |  | **Sentiment** | **Mentions** |
| National | National |  | Big 10 | Pac 12 |
| Big 12 | Big 10 |  | Pac 12 | ACC |
|  | Big 12 |  | SEC |  |
|  | SEC |  | ACC |  |
